# Duplex Nucleation and Its Effect on the Grain Size and Properties of Near Eutectic Al-Si Alloys

**DOI:** 10.3390/ma15072507

**Published:** 2022-03-29

**Authors:** Wenbo Li, Yuying Wu, Yongjie Wu, Yang Li, Amanisha Ehti, Xiangfa Liu

**Affiliations:** Key Laboratory for Liquid-Solid Structural Evolution and Processing of Materials, Ministry of Education, Shandong University, Jinan 250061, China; 17854160558@163.com (W.L.); wyj18054578314@163.com (Y.W.); liyangsdu@163.com (Y.L.); amannisaa@163.com (A.E.); xfliu@sdu.edu.cn (X.L.)

**Keywords:** Al-Si alloy, primary Si, duplex nucleation, tensile strength

## Abstract

Duplex nucleation and its effect on the grain size and properties of near eutectic Al-Si alloys were investigated in this work. It is found that the grain size of Al-13Si-1Cu alloy can be greatly refined by addition of Al-2Ti-0.5B-0.5C and Al-P master alloys, and TiB_2_/AlP duplex nucleation was observed at the nuclei of primary silicon in the process of studying the refinement mechanism. TiB_2_ phase coated by Al co-existed with AlP in the nuclei of primary Si. The existence of Al phase should not only guarantee the promotion of TiB_2_ particles on the nucleation of AlP particles, but also made TiB_2_ particle stable in the core of primary silicon through the interaction with the Si phase. Duplex nucleation can not only affect the grain size of Al-Si alloy, but also effect of the distribution of strengthening phases in Al-Si alloy and improve the properties. Compared with single Al-P master alloy treatment, the tensile strength of Al-12Si-4Cu-2Ni-1Mg alloy after duplex nucleation treatment are improved obviously. The fatigue performance of Al-12Si-4Cu-2Ni-1Mg alloy is also improved significantly by the duplex nucleation.

## 1. Introduction

Aluminum alloys are increasingly used in the automotive industry. Since its excellent wear resistance and corrosion resistance, low thermal expansion coefficient, high elevated-temperature strength and specific strength, Al-Si alloys were used to produce automobile engine components such as cylinder blocks, cylinder heads and pistons [1,2,3,4,5,6,7]. Alloying and fine grain strengthening are common strengthening methods for casting Al-Si alloys. The alloying is adding Cu, Mg, Ni and other alloying elements to form complex intermetallic phases such as Al_2_Cu, Mg_2_Si, Al_3_Ni, Al_3_CuNi and Al_7_Cu_4_Ni to improve the mechanical properties of Al-Si alloys [5,8,9], especially the high temperature performance. The fine grain strengthening improves the mechanical and surface properties of the alloy.

The refinements of Al-Si series alloys include the refinements of Al dendrite and primary silicon. The grain refinement is closely related to the two factors mentioned above. AlP can be used as the heterogeneous nuclei of primary silicon [10,11,12], as well as Al-Ti-B/Al-Ti-C intermediate alloys were often used to refine aluminum dendrites. Xiangfa Liu et al. reported an Al-2Ti-0.5B-0.5C intermediate alloy [13], which has a better refinement effect on silicon containing hypoeutectic aluminum alloy. Several experiments have found duplex nucleation [14,15,16,17,18,19,20,21], especially at the core of primary silicon. L.N. Yu et al. [18,19] found that TiB_2_ or Al_4_C_3_ particles can absorb AlP particles and have a peritectic-like coupling with them, forming a large number of coupling compounds that serve as heterogeneous nucleating substrates for primary silicon grains. Wu, Y. Y et al. [20] found that even when P content is lower than the AlP nucleation threshold concentration, AlP can nucleate on the surface of AlB_2_ to form a catalytic AlP layer, and finally primary silicon nucleate. Wang, K. et al. [21] found that AlP particles combined with γ-Al_2_O_3_ particles had enhanced the nucleation ability and efficiency of Al-P-O master alloy and promote the refinement of primary Si, and γ-Al_2_O_3_ nanoparticles could modify eutectic Si by inducing TPRE(twin plane re-entrant edge) poisoning.

However, most of the studies focus on the effect of duplex nucleation on primary silicon, but do not pay attention to the effect of duplex nucleation on grain refinement of near-eutectic Al-Si alloys. The grain refinement of materials has an important effect on their properties, especially plasticity and fatigue properties, but it is difficult to refine the grain of near-eutectic Al-Si alloys. In this work, the grain size of the near eutectic Al-Si alloy was refined by 75.2% by duplex nucleation. We will also study the duplex nucleation and its effect on the grain size and properties of near-eutectic Al-Si alloys by addition of Al-2Ti-0.5B-0.5C [22] and Al-P master alloys. The mechanism of duplex nucleation will be analyzed by focusing ion beam scanning electron microscopy (SEM) and high resolution transmission electron microscopy (HTEM).

## 2. Experimental Materials and Methods

### 2.1. Material Preparation

The alloys prepared and analyzed in the experiment were Al-13Si-1Cu and Al-12Si-4Cu-2Ni-1Mg (all compositions are in wt.% unless otherwise stated), and the materials used in this work were pure aluminum (99.7%), silicon (99.7%), pure magnesium (99.97%), copper (99.7%) and nickel (99.8%). The master alloy used in the experiment were Al-3.5P and Al-2Ti-0.5B-0.5C (provided by Shandong Al and Mg Melt Technology Co. Ltd., Jinan, China).

Firstly, Al-13Si-1Cu and Al-12Si-4Cu-2Ni-1Mg alloys were prepared in a 5 kW resistance furnace. After the temperature of melt stabilized, 0.7% C_2_Cl_6_ was added at 780 °C for degassing and slag removal, then, placed the alloy in the resistance furnace and hold for 15 min at 760 °C. Stirring master alloy after adding 1.5% Al-3.5P and holding for 30 min at 760 °C, the melt was divided into four parts and poured into four graphite crucible. 0%, 1.0%, 1.5%, and 2.0% Al-2Ti-0.5B-0.5C master alloy were added, respectively. After holding for 5 min, the melt was poured into molds. Al-13Si-1Cu alloy was poured into KBI ring iron mold and rectangular cast iron mold (The molds were preheated to 270 °C) to obtain four groups of samples A1, A2, A3 and A4. Al-12Si-4Cu-2Ni-1Mg alloy was poured into the rectangular cast iron mold and the tensile test bar mold (The preheating temperature of the mold was the same as above) to obtain four groups of samples B1, B2, B3, B4. Among them, the size of the rectangular cast iron mold is 30 mm × 170 mm × 40 mm, and the KBI ring mold has a certain slope, in which the maximum inner diameter is 55 mm, the minimum is 52 mm, and the height of the mold is 25 mm.

### 2.2. Material Characterization

The chemical composition of samples was determined by an Inductively Coupled Plasma-Atomic Emission Spectrometer PE8000 machine (MAXx, Spectro, Kleve, Germany), and the sample composition was shown in Table 1.

The A1, A2, A3, A4 alloys were etched with 0.4% HF, and the change of samples’ grain size with the addition of Al-2Ti-0.5B-0.5C master alloy was observed. The metallographic samples were taken from the same position of the strip samples and mechanically polished according to the program. The microstructure of sample was observed by optical microscope (LMCG, Wetzlar, Germany) and the field emission scanning electron microscope (FESEM, SU-70,Hitachi, Tokyo, Japan) with an energy dispersive spectroscopy (EDS, EX-250, Horiba, Kyoto, Japan) detector at 15 kV. Focused ion beam scanning electron microscopy (Lyra 3 XMU, TESCAN, Brno, Czech Republic) was used to analyze the element distribution and phase composition of particles in the samples.

The B1, B2, B3 and B4 alloy test bars were processed into standard “dog-bone” type specimens. The specimens were subjected to T6 heat treatment under the following conditions: solution treatment at 510 °C for 2 h and aging treatment at 180 °C for 8 h. The fatigue life of the specimens after heat treatment was measured by PWS-50 electro-hydraulic servo dynamic and static universal testing machine under different stress amplitudes. Hardness blocks with a thickness of 10 mm were taken from the B1, B2, B3 and B4 alloy strip samples, and the blocks were subjected to T6 heat treatment under the same conditions. In addition, the hardness of the obtained samples was measured by a digital Brinell hardness tester (HBST-3000AET, Huayu, Yantai, China).

Samples with a size of 1 mm × 1 mm × 1 mm were cut from the tensile test bars of B1 and B4 alloys, The internal microstructure of the samples was characterized by microscopic CT (SkyScan2211, Bruker, Antwerp, Belgium) with a resolution of 200 nm, and the volume, quantity and connection degree of the phase to be measured inside the sample were calculated and analyzed by software. The Al-2Ti-0.5B-0.5C master alloy was characterized by the field emission scanning electron microscope with an energy dispersive spectroscopy detector at 15 kV.

## 3. Result and Discussion

### 3.1. Effect of Duplex Nucleation on the Grain Size and Microstructure of Near Eutectic Al-Si Alloy

**Figure 1:**
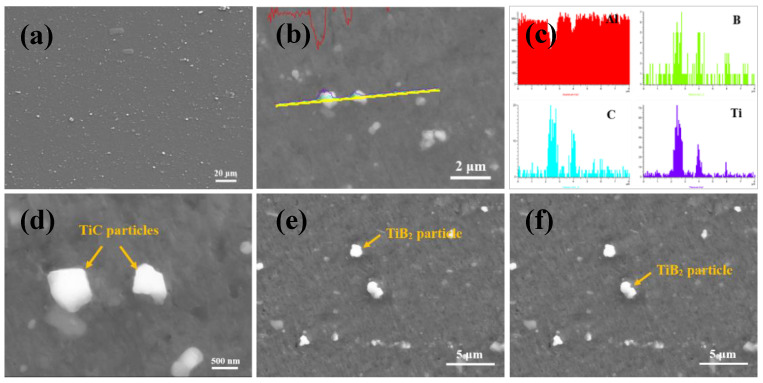
(**a**) Morphology of Al-Ti-C-B master alloy, (**b**–**d**) TiC particles, (**e**,**f**) TiB_2_ particles.

Al-Ti-C-B master alloy was characterized and the microstructure of the master alloy was obtained as shown in Figure 1. It can be seen from the figure that the Al-Ti-C-B master alloy contains TiB_2_ and TiC particles with sub-micron size and uniform distribution in the master alloy. The Al-3.5P master alloy used in the experiment is composed of aluminum matrix and AlP particles [23].

**Figure 2:**
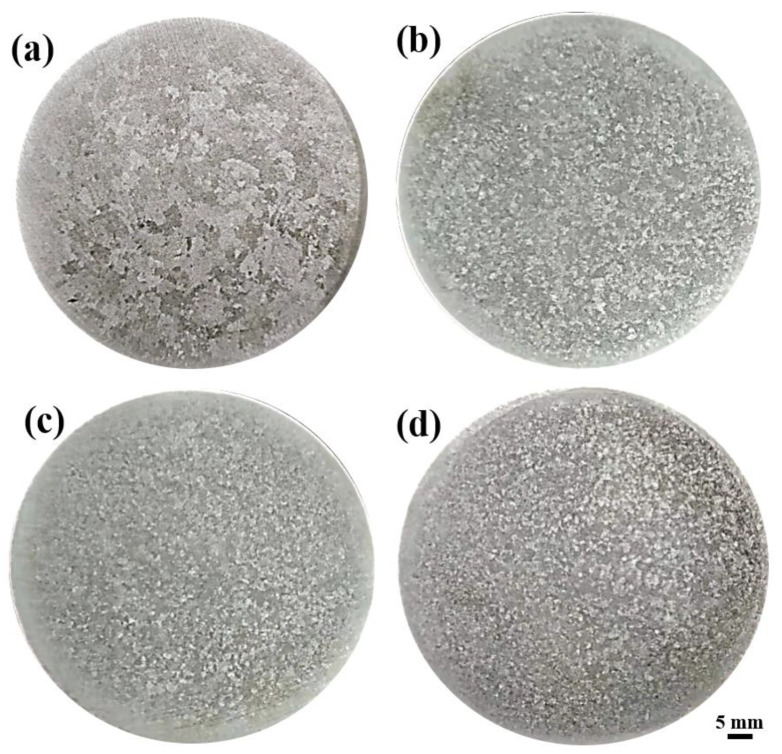
Grain image of Al-13Si-1Cu alloy with different addition amounts of Al-2Ti-0.5B-0.5C master alloy: (**a**) A1 alloy, (**b**) A2 alloy, (**c**) A3 alloy, (**d**) A4 alloy.

**Figure 3:**
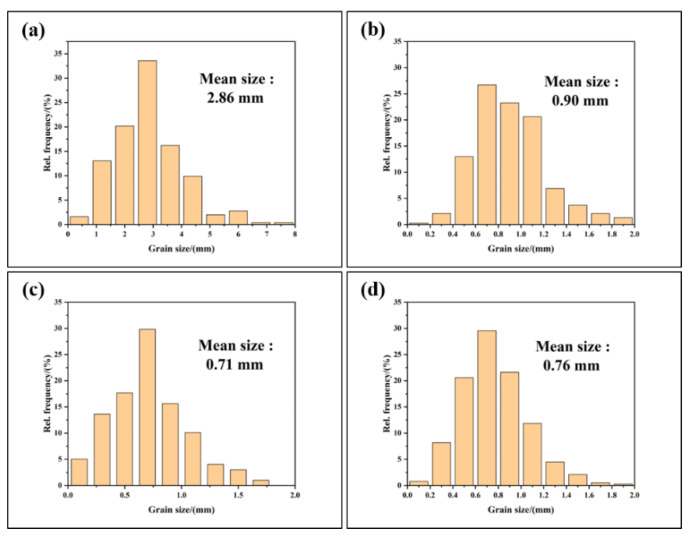
Statistics of grain size of Al-13Si-1Cu alloy with different addition amounts of Al-2Ti-0.5B-0.5C master alloy: (**a**) A1 alloy, (**b**) A2 alloy, (**c**) A3 alloy, (**d**) A4 alloy.

In order to study the effect of Al-2Ti-0.5B-0.5C master alloy on grain size of Al-13Si-1Cu alloy, A1, A2, A3 and A4 samples were etched by 0.4% HF. Figure 2 shows the different samples after being etched. It can be seen from the Figure 2 that the grain size decreases after the addition of Al-2Ti-0.5B-0.5C master alloy, indicating that Al-2Ti-0.5B-0.5C master alloy has a refining effect on Al-13Si-1Cu alloy. Nano Measure software was used to make statistics on the grain size of the alloy, and the grain size distribution of the four gold combinations was obtained as shown in Figure 3. It can be seen from the figure that when the addition amount of Al-2Ti-0.5B-0.5C master alloy is 1.5%, the grain size of the alloy is the finest, and this amount has the best refining effect.

**Figure 4:**
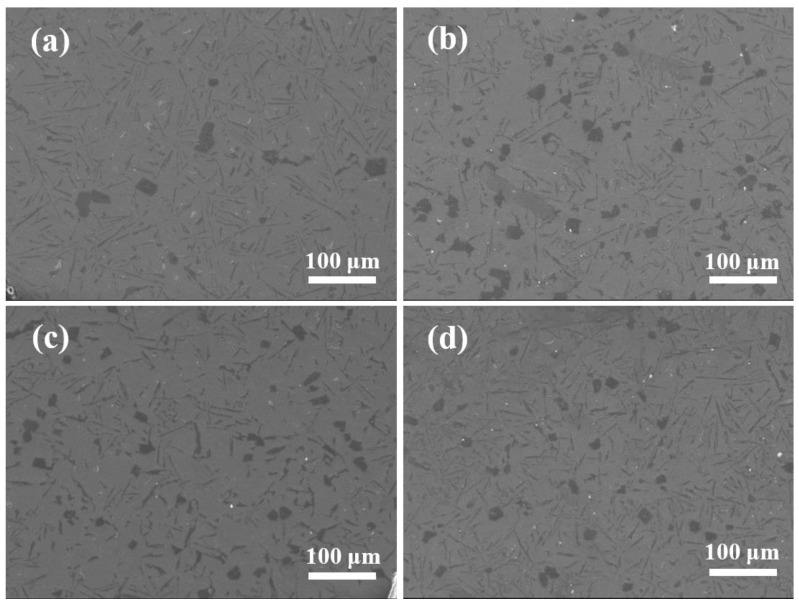
Microstructure diagram of Al-13Si-1Cu alloy with different addition amounts of Al-2Ti-0.5B-0.5C master alloy: (**a**) A1 alloy, (**b**) A2 alloy, (**c**) A3 alloy, (**d**) A4 alloy.

Figure 4 is the SEM image shows the microstructure of the four samples. It can be seen from the figure that after the addition of Al-2Ti-0.5B-0.5C master alloy in the alloy, the precipitation amount of primary silicon increases. As shown in Figure 4c, the effect was most obvious when the addition amount was 1.5%, compared with Figure 4a, the amount of primary silicon increases by about 4.4 times and the size of primary silicon decreases by about 47%.

**Figure 5:**
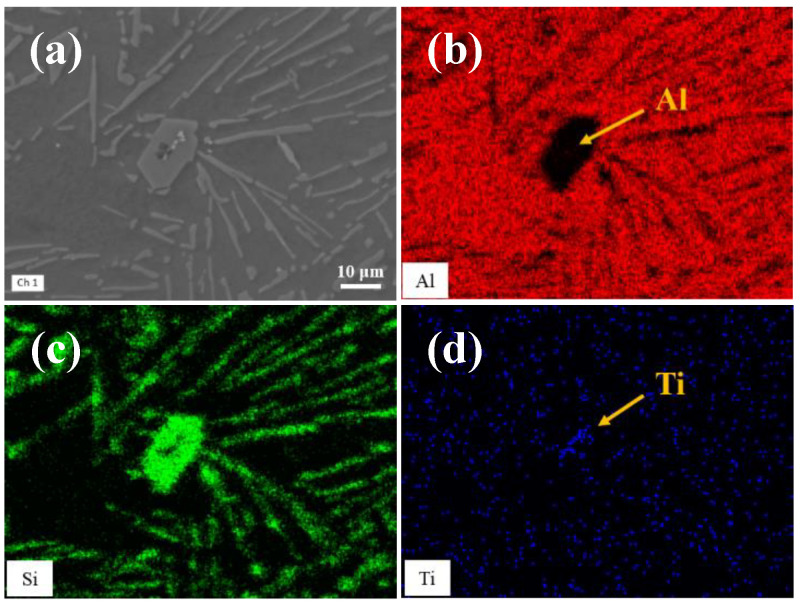
Element distribution at the primary silicon of the A2 alloy. (**a**) Morphology of primary Si, (**b**) Al element, (**c**) Si element, (**d**) Ti element.

The microstructure of the alloy was further analyzed by scanning electron microscopy (SEM). As shown in Figure 5, different phases were found in the core of primary silicon of A2 alloy. The enrichment of element Ti and Al was found by surface scanning analysis. The TiC and TiB_2_ particles can promote the nucleation of primary silicon with AlP through the duplex nucleation [18,19], but scanning electron microscopy alone can’t tell what the nucleation core is.

**Figure 6:**
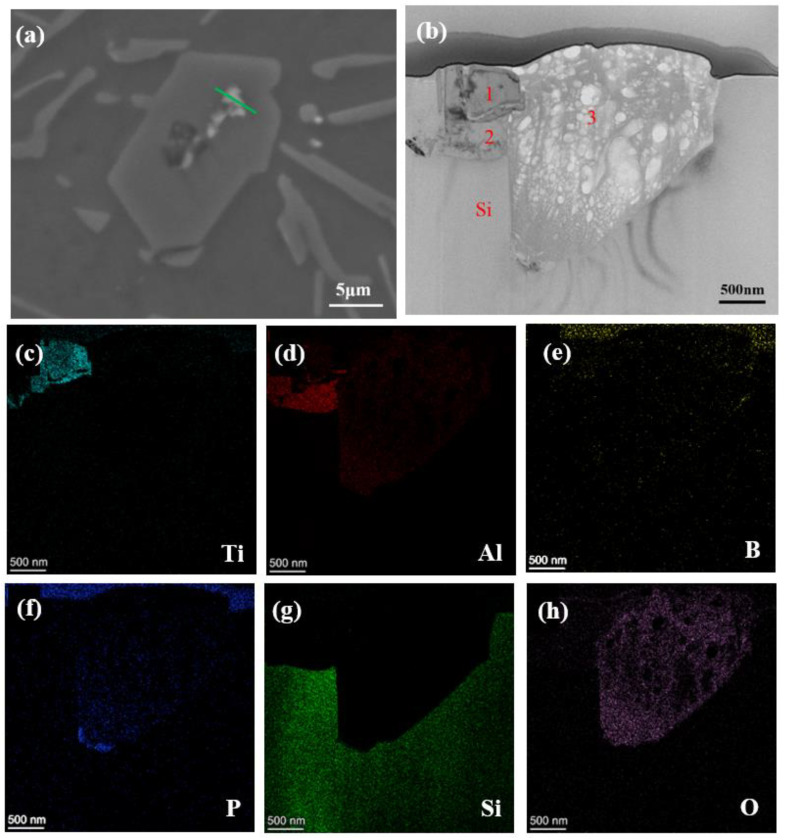
FIB analysis of the nucleation site of primary Si (**a**) cutting position, (**b**) section morphology, (**c**–**h**) HADDF diagram of the section.

To investigate the duplex nucleation further, focused ion beam (FIB) was used to cut the core of primary silicon, and the section was characterized by scanning electron microscopy. The cutting position and section morphology were shown in Figure 6a,b. From the morphology diagram, it could be seen that there were three kinds of phases in the core of primary silicon, which indicated that the nucleation of primary silicon was the result of the interaction of multiple phases. Figure 6c–h shows the element distribution at the section. It can be seen from the figure that phase 1 is composed of element Ti and B, phase 2 is composed of Al element, and phase 3 is composed of Al element, P element and O element.

**Figure 7:**
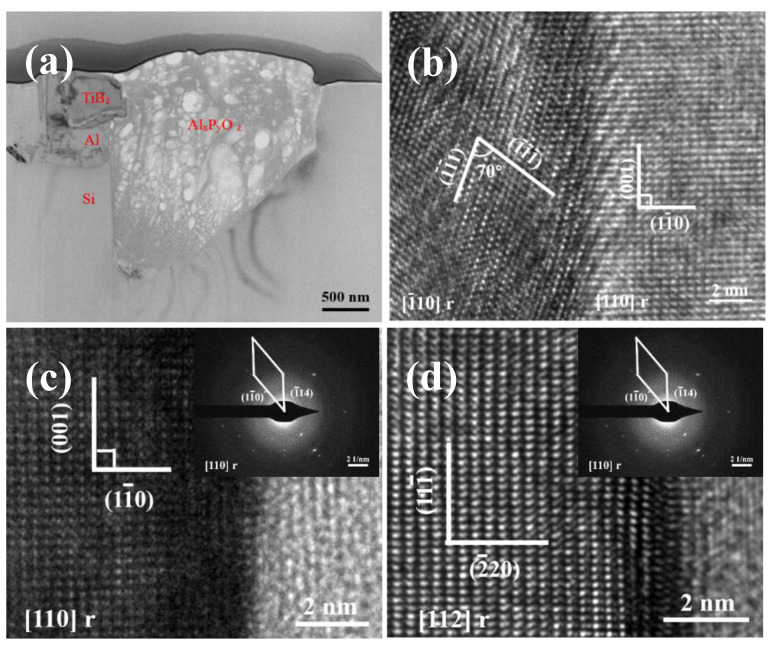
HRTEM diagram of interface in the primary Si (**a**) Section morphology of primary Si, (**b**) Al-TiB_2_ interface, (**c**) TiB_2_-Al_x_P_y_O_z_ interface, (**d**) Si-Al_x_P_y_O_z_ interface.

In order to further confirm the composition of the phases, high resolution transmission electron microscopy (HRTEM) was used to characterize the interfaces between these three phases, as shown in Figure 7. Combining element distribution and high-resolution analysis, phase 1 is TiB_2_, phase 2 is Al, and phase 3 is Al_x_P_y_O_z_. High resolution interface display that all of Al/TiB_2_, TiB_2_/Al_x_P_y_O_z_ and Si/Al_x_P_y_O_z_ have good interface relations. For the sources of Al_x_P_y_O_z_, combined with the addition of Al-3.5P master alloy in the experimental process, it should be that the AlP particles obtained after the addition of master alloy hydrolyzed in the mechanical polishing process, and finally obtained the Al_x_P_y_O_z_ multicomponent compound.

**Figure 8:**
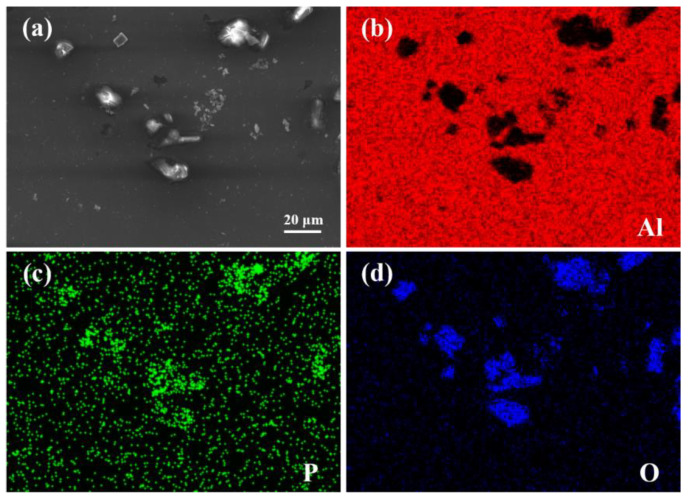
EDS composition of Al-3.5P master alloy (**a**) Morphology of Al-3.5P alloy, (**b**) Al element, (**c**) P element, (**d**) O element.

In order to verify this speculation, we conducted mechanical polishing on the Al-3.5P master alloy and observed it under the scanning electron microscopy as shown in Figure 8. It can be seen that the AlP particles contained in the Al-3.5P master alloy hydrolyzed during sample treatment to obtain Al_x_P_y_O_z_ multicomponent compounds, which confirmed our previous speculation.

**Figure 9:**
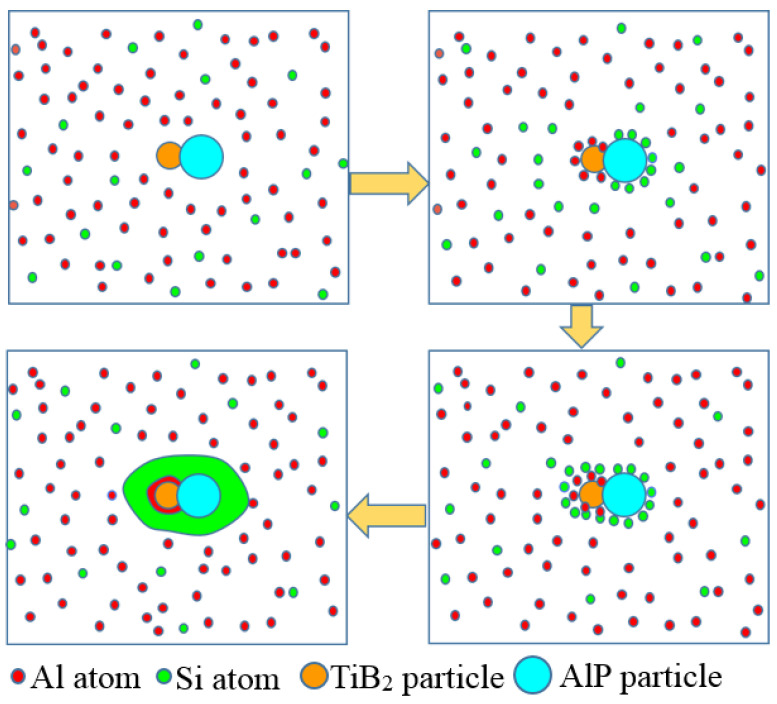
Schematic description of the duplex nucleation mechanism of AlP and TiB_2_ in the near-eutectic Al-Si alloy.

In previous studies, AlP can be used as the nucleation core of primary silicon [10,11,24], and TiB_2_ can be coupled with AlP as the nucleation core of primary silicon through peritectic coupling [19]. However, TiB_2_ particles which was observed to nucleate diphasically with AlP particles in the primary silicon core, was coated with Al phase in this work. We think Al plays a very important role in diphasic nucleation. The schematic description of the duplex nucleation mechanism of AlP and TiB_2_ in the near-eutectic Al-Si alloy is shown in Figure 9. First of all, AlP nucleates on the surface of TiB_2_ particles, Si element aggregates near AlP particles and primary silicon begins to form [11]. Al phase nucleates and grows on TiB_2_ particles [25,26], the content of Al element around the particles’ changes, and the eutectic component is reached in a small area. When the primary silicon grows near TiB_2_ particles, it continues to grow on the surface of Al phase in a manner similar to the formation of eutectic tissue [27]. Finally, the nucleation and growth of primary silicon are achieved by synergistic action of TiB_2_ and AlP particles. In the process of sample treatment, AlP hydrolyzes when it meets with water [28,29], therefore, we observe the co-existence of Al_x_P_y_O_z_ multicomponent compound and TiB_2_ particle coated by Al phase at the core of primary silicon.

Al-2Ti-0.5B-0.5C master alloy has grain refinement effect on near-eutectic Al-Si alloy. Combined with scanning electron microscopy and section analysis, TiB_2_/AlP duplex nucleation can improve the refining efficiency of AlP particles during the refining process, and Al phase plays an important role in the process of duplex nucleation. The primary silicon phase cannot directly nucleate and grow on the surface of TiB_2_ particles. However, after being coated with Al, TiB_2_ particles and AlP particles can coexist in the core of primary silicon particles. The existence of Al phase not only ensures that TiB_2_ particles promote the nucleation of AlP particles. The TiB_2_ particles can exist stably in the core of the primary silicon through the interaction with the primary silicon phase. The existence of Al phase not only guaranteed the promotion of TiB_2_ particles on the nucleation of AlP particles, but also made TiB_2_ particles can exist stably in the core of the primary silicon through the interaction with the primary silicon phase.

### 3.2. Effect of Al-2Ti-0.5B-0.5C on the Microstructure and Properties of Al-12Si-4Cu-2Ni-1Mg Alloys

For investigating the effect of Al-2Ti-0.5B-0.5C master alloy to the near-eutectic Al-Si alloy on the microstructure and properties, Al-12Si-4Cu-2Ni-1Mg was selected as the base alloy, and after modification with 1.5% Al-3.5P master alloy. The B1, B2, B3 and B4 alloys were obtained by adding 0%, 1.0%, 1.5% and 2.0% Al-2Ti-0.5B-0.5C master alloy, respectively. The microstructure changes of the alloy were analyzed and the properties of the alloy were tested.

**Figure 10:**
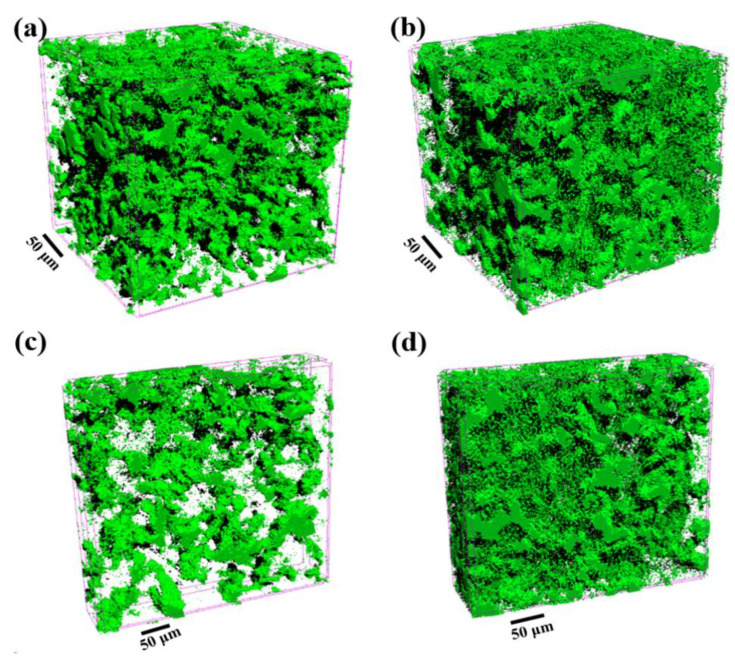
3D structure rendering of alloy phase and eutectic Si: (**a**,**c**) B1 alloy; (**b**,**d**) B4 alloy.

For the sake of studying the effect of Al-2Ti-0.5B-0.5C master alloy on the near-eutectic Al-Si alloy, the three-dimensional structure of B1 and B4 alloy was tested by microscopic CT, as shown in Figure 10. Meanwhile, software was used to calculate the characteristic values of the three-dimensional structure constructed by the alloy phase and eutectic silicon [30,31,32], the calculation results were shown in Table 2. Compared with 12.24% of the original alloy, the connectivity rate is increased by 58.3%, which indicates that the alloy phase and eutectic silicon are connected more closely after the addition of Al-2Ti-0.5B-0.5C master alloy.

**Figure 11:**
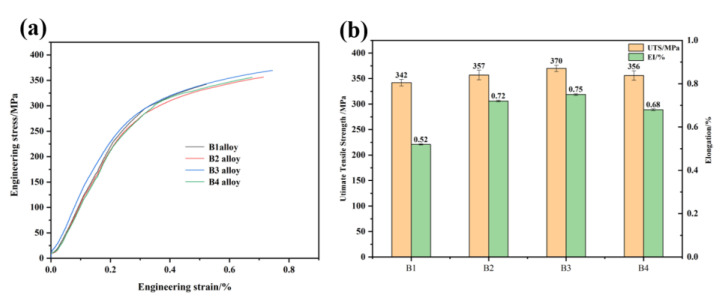
Tensile properties of Al-12Si-4Cu-2Ni-1Mg alloy at room temperature: (**a**) tensile curve; (**b**) tensile strength and elongation.

The change of microstructure will affect the properties of the alloy. Therefore, we further studied the influence of the addition of master alloy on the properties of the alloy. Figure 11 shows the tensile properties of Al-12Si-4Cu-2Ni-1Mg alloy at room temperature. Figure 11a shows the tensile curve, and Figure 11b shows the tensile strength and elongation. Without refinement, the ultimate tensile strength and elongation of Al-12Si-4Cu-2Ni-1Mg alloy at room temperature are about 342 MPa and 0.52%, respectively. After adding 1.5% Al-2Ti-0.5B-0.5C master alloy, the ultimate tensile strength and elongation of Al-12Si-4Cu-2Ni-1Mg alloy at room temperature are about 370 MPa and 0.75%, respectively. Compared with no refinement, the ultimate tensile strength of Al-12Si-4Cu-2Ni-1Mg alloy after refinement with 1.5% Al-2Ti-0.5B-0.5C master alloy increases by about 8.2% at room temperature.

**Figure 12:**
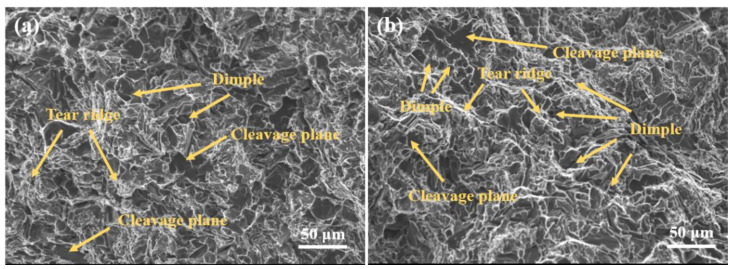
Tensile fracture at room temperature of B1 alloy (**a**), B3 alloy (**b**).

The fracture of tensile specimen at room temperature is shown in Figure 12. As can be seen from the figure, cleavage planes exist at the fracture of the sample before and after the addition of Al-Ti-C-B master alloy, and the fracture mechanism of the sample is brittle fracture. However, in comparison with the samples without refinement, after adding Al-Ti-C-B master alloy for refinement, the dimple and tear edge of the alloy increase, and the dimple becomes deeper, so the tensile strength and elongation of the alloy are improved at room temperature, but the elongation is still lower than that of other alloys. The addition of Al-Ti-C-B intermediate alloy did not change the fracture mechanism of the alloy, but changed the microstructure of the alloy, and affected the tensile properties of the alloy at room temperature.

**Figure 13:**
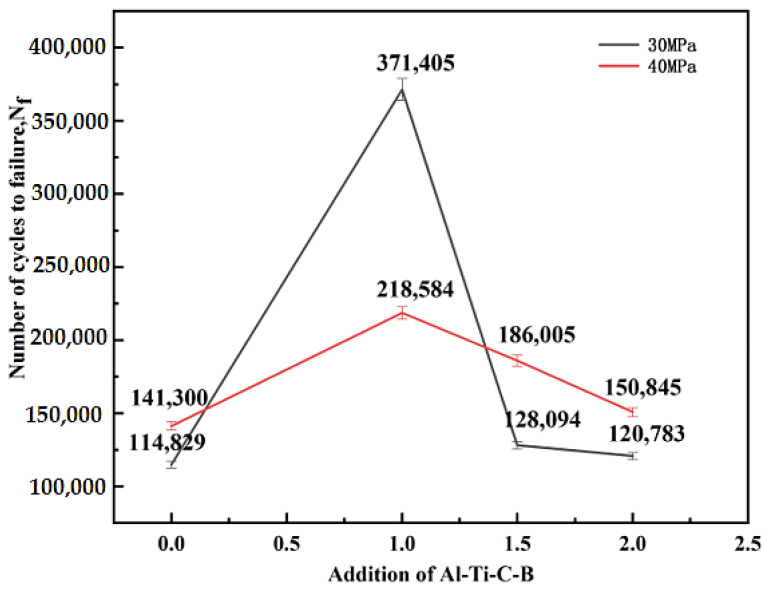
Fatigue properties of Al-12Si-4Cu-2Ni-1Mg alloy with different amounts of Al-2Ti-0.5B-0.5C master alloy.

Moreover, the addition of Al-2Ti-0.5B-0.5C master alloy has a greater effect on the fatigue properties of the alloy. As shown in Figure 13, the fatigue life of the alloy with stress amplitude of 30 MPa and 40 MPa is measured. It can be seen from the broken line chart that the alloy has the longest fatigue life when the addition amount of the Al-2Ti-0.5B-0.5C master alloy is 1.0%. Therefore, for the fatigue properties of the alloy, 1.0% is the best addition.

## 4. Conclusions

Duplex nucleation and its effect on the grain size and properties of near eutectic Al-Si alloys were investigated in this work. The effect of Al-2Ti-0.5B-0.5C master alloy on the microstructure and properties of the near-eutectic Al-Si alloy modified by Al-3.5P master alloy were investigated. The conclusions are summarized as follows:

(1) TiB_2_ particles can improve the nucleation efficiency of AlP particles by duplex nucleation with AlP particles. During the duplex nucleation process, The Al phase coating on the surface of TiB_2_ particles not only ensures that TiB_2_ particles can promote the nucleation of AlP particles, but also ensures that TiB_2_ particles can coexist with AlP particles in the core of primary silicon
(2) The Al-2Ti-0.5B-0.5C master alloy can play a role in grain refinement of near-eutectic Al-Si alloy, it can affect the three-dimensional structure constructed by the alloy phase and eutectic silicon of Al-12Si-4Cu-2Ni-1Mg, and improve the fatigue property of the alloy. After adding 0.5% Al-2Ti-0.5B-0.5C master alloy, the ultimate tensile strength and elongation of Al-12Si-4Cu-2Ni-1Mg alloy at room temperature are about 355 MPa and 0.25%, respectively. The ultimate tensile strength at 350 °C is about 89 MPa, and the elongation is about 10.5%. The fatigue property of the alloy can be greatly improved when adding 1% Al-2Ti-0.5B-0.5C master alloy.

## Figures and Tables

**Table 1:** Chemical composition of A1, B1 alloy (wt. %).

	Al	Si	Cu	Mg	Ni
A1	85.82	13.09	1.09	0	0
B1	79.88	13.32	3.56	0.91	2.33

**Table 2:** Eigenvalues of 3D network structure of alloys.

Alloys	Connectivity Rate I (%)	Euler Number Χ	Structure Thickness Φ (μm)
B1	12.24	26,602	5.03
B4	19.38	75,132	6.04

## Data Availability

Not applicable.
